# Mental Health and Quality of Life in Pulmonary Embolism: A Literature Review

**DOI:** 10.3390/arm91020015

**Published:** 2023-04-20

**Authors:** Niki Gkena, Paraskevi Kirgou, Konstantinos I. Gourgoulianis, Foteini Malli

**Affiliations:** 1 Respiratory Disorders Lab, Faculty of Nursing, University of Thessaly, Gaiopolis, 41500 Larissa, Greece; 2 Respiratory Medicine Department, Faculty of Medicine, School of Health Sciences, University of Thessaly, Biopolis, 41110 Larissa, Greece

**Keywords:** pulmonary embolism, mental health, quality of life, QoL assessment tools, anxiety, depression, post-traumatic stress disorder

## Abstract

**Highlights:**

**What are the main findings?**

- The mental health impacts of an acute episode of pulmonary embolism include post-traumatic stress disorder, anxiety and depression that may last up to 2 years after the diagnosis.
- Quality of life is reduced during the first months of the acute episode, and patients display an improvement during follow-up.

**What are the implications of the main findings?**

- Evaluating mental health and quality of life in PE patients during follow-up may be clinically important.
- Development of an assessment strategy to adequately address mental health of PE patients may be useful.

**Abstract:**

Pulmonary embolismis an acute disease with chronic complications and, although it is not considered a chronic disease, it requires close follow-up. The scope of the present literature review is to decode the existing data concerning quality of life and the mental health impact of PE during the acute and long-term phases of the disease. The majority of studies reported impaired quality of life in patients with PE when compared to population norms, both in the acute phase and >3 months after PE. Quality of life improves over time, irrespectively of the measurement used. Fear of recurrences, elderly, stroke, obesity, cancer and cardiovascular comorbidities are independently associated with worse QoL at follow-up. Although disease specific instruments exist (e.g., the Pulmonary Embolism Quality of Life questionnaire), further research is required in order to develop questionnaires that may fulfil international guideline requirements. The fear of recurrences and the development of chronic symptoms, such as dyspnea or functional limitations, may further impair the mental health burden of PE patients. Mental health may be implicated by post-traumatic stress disorder, anxiety and depressive symptoms present following the acute event. Anxiety may persist for 2 years following diagnosis and may be exaggerated by persistent dyspnea and functional limitations. Younger patients are at higher risk of anxiety and trauma symptoms while elderly patients and patients with previous cardiopulmonary disease, cancer, obesity or persistent symptoms exhibit more frequently impaired QoL. The optimal strategy for the assessment of mental health in this patient pool is not well defined in the literature. Despite mental burden being common following a PE event, current guidelines have not incorporated the assessment or management of mental health issues. Further studies are warranted to longitudinally assess the psychological burden and elucidate the optimal follow-up approach.

## 1. Introduction

Pulmonary embolism is one of the two forms of venous thromboembolism (VTE). Frequently, PE has multifactorial causes, although some patients do not have any identifiable risk factors [1,2]. When left undiagnosed and, thus, untreated, PE can be lethal in as much as 30% of sufferers [3,4]. High-risk patients presenting with hemodynamic instability may require immediate reperfusion therapy, Intensive Care Unit (ICU) admission and even extracorporeal membrane oxygenation (ECMO) to prevent death [3,5,6]. Thus, some patients experience PE as a life-threatening event that requires aggressive treatment, while some describe PE as a life-changing event. On the other hand, low risk patients may be treated as outpatients with acceptable safety [7,8]. The benefit of outpatient management in terms of quality of life is still under debate with some studies reporting reduced rates of anxiety and increased comfort when treated as outpatients [9].

Patients with chronic diseases frequently report emotional and behavioral problems, as well as poor mental health [10]. PE is an acute disease with chronic complications, and, although it is not considered a chronic disease, it requires close follow-up [11]. The long-term medical consequences of PE have been studied previously in the literature and may include persisting or deteriorating dyspnea and poor physical performance, which are frequently present 6 months to 3 years after an acute PE episode and may be signs of post-PE syndrome [12]. 

Chronic thromboembolic pulmonary hypertension (CTEPH) is a rare but devastating complication after acute PE. The current guidelines are focused on the physical outcomes of PE and disregard the mental consequences of the disease [3,5]. Studies have shown that PE diagnosis is accompanied by mental distress derived from the lack of adequate information and the fear of further complications. Although this distress is not unusual, it is frequently overlooked [13]. The increased mental burden following PE diagnosis is underlined by the increased use of psychotropic drugs following a venous thromboembolism diagnosis [10,14].

The scope of the present literature review is to decode the existing data concerning quality of life and the mental health impacts of PE during the acute and long-term phases of the disease.

## 2. Literature Search

We conducted a comprehensive literature search for English language publications using PubMed, MEDLINE and Google Scholar. The following search strategy combinations were used: (quality of life OR stress OR post-traumatic stress OR anxiety OR well-being OR mental health OR psychological) AND (pulmonary embolism OR outpatient care) and (QoL assessment tools OR SF-36 OR PEmbQoL OR SF-12). We did not impose any type of study restrictions. We found 1396 articles but only 134 were relative to our aim. Then, two researchers, Foteini Malli and Niki Gkena removed duplicates and studies that did not address our aim. After reading these papers thoroughly, we included only those with content relevant to mental health and quality of life after experiencing a PE and those that we evaluated as important for consideration in the review. Furthermore, FM and NG manually searched the references from the articles identified.

## 3. Mental Health Aspects of pulmonary embolism

Mental health is defined as a state of well-being in which one comprehends one’s own abilities, effectively manages the everyday stress of life, works productively and benefits one’s community [5]. The association of mental and physical health is bidirectional; subjects with mental illness are at increased risk of sleep disorders, diabetes, heart disorders and/or stroke [15] while patients with chronic medical conditions have a higher prevalence of mental health disorders [16,17]. Impairment of mental health status in chronic respiratory patients results in reduced health-related outcomes, lower adherence to pharmacological interventions [18] as well as lower quality of life [19]. Taking the aforementioned concepts into consideration, it seems imperative to address mental health status in chronic patients in order to improve their physical outcomes.

Quality of life (QoL) is defined as a persons’ perception of their position in life after taking into account their culture and value systems [20]. Health-related (HR) QoL refers to a patient’s self-reported impact of the disease and treatment on the subjects physical, psychological and social functioning. A large number of clinical studies have demonstrated that QoL presents an outcome measure that corresponds to clinical changes [16]. Mental health is an important element of QoL, and the two conditions are interrelated. For example, depression, which is an important factor of mental health, may worsen HRQoL, since it may further impair the perception of somatic symptoms and decrease general functioning [21].

Pulmonary embolism is associated with troublesome and worrying symptoms, such as acute onset dyspnea, hemoptysis and/or loss of consciousness, while the disease is life-threatening [3]. As discussed earlier, acute PE treatment includes medical as well as invasive therapies, while long-term and extended treatment commonly includes anticoagulants that may complicate the course of the disease with bleeding [3]. The aforementioned data, in conjunction with the fear of recurrences and the post-PE syndrome, among others, explain why PE is considered a health-related crisis, both in the acute as well as in the long-term phase, with potential implications in mental health and especially QoL [22,23].

### 3.1. Quality of Life in PE Patients

Impaired HRQoL has been associated with increased mortality in the general population and in subjects with chronic diseases [17,18]. A significant number of instruments have been developed in order to assess a patient’s view of their functional status and QoL [19]. One of the most popular instruments is the 36-Item Short Form Health Survey questionnaire (SF-36), which evaluates HRQoL [24]. The EuroQol Group 5 Dimensions 5 Levels (EQ-5D-5L) is a standardized generic measure of QoL in assessing population health and is one of the most popular multi-attribute utility instruments used in clinical and economic evaluations [25]. 

Although the SF-36 has been used in the assessment of HRQoL, there was the need of a disease-specific questionnaire with high sensitivity in detecting and quantifying the impact of an acute PE in HRQoL. The first and only disease- specific questionnaire is the Pulmonary Embolism-Quality of Life (PEmb-QoL) questionnaire [26,27]. A systematic review assessing the methodology and development of instruments about HRQoL questionnaires for patients with venous thromboembolism concluded that none of the available instruments fulfill the guideline recommendations and, due to other methodological limitations, their use in everyday practice has limitations [28].

We describe the main findings of QoL changes following PE in the acute phase (first 3 months) and at long-term (>3 months).

#### 3.1.1. HRQoL during the First 3 Months after Diagnosis (Acute Phase)

Limited data exist on QoL of PE patients in the acute phase of the disease. A multicenter prospective cohort study (ELOPE) described HRQoL based on SF-36 and PEmb-QoL instruments; dyspnea and functional exercise capacity after a first episode of acute PE where the most important components of patients’ QoL after an acute PE [29]. According to the ELOPE cohort study, these components improve during follow-up, most notably during the first 3 months after diagnosis and patients display a meaningful improvement [30]. However, female sex, higher BMI and exercise limitation on 1-month cardiopulmonary exercise test (percent-predicted VO2 peak <80%) are clinical and physiological predictors of decreased improvement [30].

In an interventional multicenter trial (HoT-PE), QoL was assessed after early discharge in low-risk PE [31]. Patients were treated with rivaroxaban. The participants completed standardized, disease-specific and generic quality of life questionnaires (PEmb-QoL, EQ-5D-5L, Anti-Clot treatment scale) at 3 weeks and 3 months after the diagnosis [31]. All results showed an early improvement in quality of life during follow-up. Similarly to the ELOPE study [29], female sex, increased BMI and a history of cardiac or pulmonary disease were associated with poorer QoL [32].

The effect of the type of therapy on HRQoL during the first 3 months has not been extensively studied. Kline et al. [33] reported that patients treated with systemic thrombolysis exhibited a better SF-36 physical component score, but not mental score, versus patients treated with placebo. A potential criticism of the aforementioned findings is that the study included normotensive patients and that it was terminated prematurely.

#### 3.1.2. HRQoL after 3 Months of Acute PE Diagnosis (Long Term)

Several studies have examined HRQoL of PE patients during the long term and extended phase of therapy; most of the studies reported impaired QoL that improves over time (Table 1). The selection of studies included in Table 1 are based on the use of adequate QoL measurements and the report of clear results of PE impacting in QoL and mental health. Researchers have demonstrated that HRQoL assessed by EQ-5D-5L is worse when compared to the general population at >3 months since the diagnosis [25,34]. Similar results are obtained when HRQoL is measured with PEmbQoL [24,30] or SF-36 [30,35]. Irrespectively of the instrument used, HRQoL seems to improve over time [24,34,36]. The latter observations suggest a possible negative role of functional limitations in QoL. As far as recurrences, studies have inconsistent results. Some researchers have not associated recurrences with worse QoL [34], although others have demonstrated that recurrent VTE is an independent predictor of some aspects of HRQoL [35].

No existing prediction model can adequately predict HRQoL after PE [35]. Interestingly, qualitative data suggest that the fear of recurrence is linked to impaired HRQoL [37]. Other factors that patients refer to worse HRQoL include restriction of social life and hobbies [37]. Data suggest that age [34,35], previous stroke [34], obesity, active malignancy and cardiopulmonary disease [35] are independent predictors of HRQoL. On the other hand, longer disease-free interval and PE provoked by transient risk factors may be independent predictors of better HRQoL [35]. The latter may be underlined by the frequent stop of anticoagulation following a PE associated with reversible risk factors [3]. Data about whether the type of drug therapy (DOACs or Vit K antagonists) may influence QoL are conflicting. Some studies report that QoL is better in patients receiving DOACs over Vit K antagonists [38], while others challenge this finding [39]. The discrepancies among studies may be attributed to the differences in sample size among studies.

### 3.2. Post-Traumatic Stress Disorder (PTSD)

The impact of PE in the patients’ psychology is prevalent. Several studies have observed high levels of PTSD in PE patients [40]. After experience a PE, especially major/massive PE, patients refer to it as a life-changing event with some of them reporting a loss of identity and role in life [40]. The feeling of upcoming death that accompany the diagnosis of PE often creates a post-traumatic fear that the same symptoms will recur during treatment period or at any time during their lives [40,41]. This results in constant fear and psychological distress. In particular, adolescents and young adults with PE complain about the possibility of a health threat in the future and/or a potential risk of dying of a recurrent event [30]. Younger patients report a greater emotional impact of VTE and may require heightened level of support [30,40].

The role of health care professionals is to identify and support patients at risk of post-traumatic stress and help them reduce distress by targeting at enhancement of a psychological wellbeing. The main areas where health care professionals can give support to PE patients include information regarding their condition, prognosis and long-term treatment; empathy; early identification of psychological distress; and early intervention [40]. Scarce data exist about the optimal intervention that could improve PTSD following PE, but researchers have suggested that a combination of early identification of patients at risk, psychoeducation and post traumatic counselling may benefit the patient’s population [40]. One must acknowledge that, if health care professionals focus solely on clinical outcomes (VTE recurrence, bleeding and drug adverse effects), they will not be able to efficiently address the mental distress of their patients [42].

### 3.3. Anxiety Disorders

High levels of anxiety are observed after unexpected acute illness, especially following hospital discharge [40]. According to the results of different studies exploring psychological impact and patients’ perception after an acute PE, all patients expressed major or minor psychological distress [43]. Patients reported being forever changed by the experience with physical and psychological reminders of PE [43]. High levels of trauma and anxiety symptoms are the most common expression of PE’s impact [44]. Most patients expressed that everyday life is influenced negatively and report intense worries of not being able to notice symptoms of a recurrent PE in time [44]. Others fear that, if they used the health care system, their symptoms would be underestimated and would not be taken seriously. 

Patients report that experiencing anxiety during their hospital stay influenced their psychology. They were asked to “wait and see”, underwent differential diagnostic examinations that delayed diagnosis and the initiation of therapy [32]. Patients express that due to the acute nature of the disease, the amount of information received and the examination performed in only a few days, their ability to fully understand and express their feelings and inquiries to the health care professionals is impaired [44,45]. The lack of continuous communication with their physician, the uncertainty after stopping the anticoagulant treatment, the incomplete information about their follow-up and the fear of recurrence made them feel frustration and insecurity [45].

Factors that have been associated with anxiety, even at 2 year follow-up after PE, include persistent dyspnea and everyday life limitations [46]. Age may affect feelings of anxiety following PE diagnosis, with younger patients being more at risk. Younger patients report fear of losing their independence and resting on their families and on the health care system [43]. Older patients referred that their life experience helped them accept the situation more easily [43]. 

Both age groups report that there is a lack of knowledge in the primary health care system and they fear of not being accepted in time in case of recurrence [43]. One must take into account that until the previous decade, the only oral treatment of PE was Vit-K antagonists, which were associated with increased bleeding events and a narrow therapeutic window [47]. Therefore, stress about bleeding and repeated withdrawal for checking the international normalized ratio (INR) caused additional anxiety and possibly more emergency department visits.

Concerning their family and social environment, patients seem to have trouble being involved as they did before suffering from PE [44]. They reported that their families and friends must make an extra effort to maintain the daily routine and social life as they do not feel calm and foul of energy [32]. For example, some patients fear traveling abroad, participating in social events and having lunch/dinner with friends because of their changed food and drink habits. Therefore, they feel more fragile and sensitive, are worried and ruminating, blame themselves for their previous habits and think that the additional burden they put on their families create a general distress and anxiety state [48].

### 3.4. Depression and Depressive Symptoms

Studies report that patients develop psychological symptoms (including depression, intrusion and increased scrutiny of the body) after an acute PE, that may last up to 2 years, in some cases [32]. Depressive symptoms may affect more than 50% of patients. A rate of 69% of PE patients experiences traumatic sensations of reliving the thrombotic event, such as flashbacks, nightmares and panic attacks. Approximately 40% of PE survivors complain about disordered impulse control and maladjustment, being angry or ashamed with their bodies and feel unattractive. These symptoms decrease productivity, body functionality, feeling of good health and lead to social isolation and, occasionally, to loss of employment [32].

The pathophysiological and psychological responses of an acute illness, such as PE, are simply an emergent stressor for suffering patients and lead to anxiety and depression [49]. Dyspnea and severe arterial hypoxemia, such as in other pulmonary diseases, can solely increase the depression and PaO2 is an independent predictive factor for depression establishment [49]. Until the previous decade, the only oral treatment of PE was Vit-C antagonists, which were associated with increased bleeding events and narrow therapeutic window [47]. Therefore, stress about bleeding and repeated withdrawal for checking the international normalized ratio (INR) caused additional anxiety and lead to the emergencies. Patients at high-risk PE tend to experience more depressive symptoms than those in low risk of early death [49]. Younger PE patients report depression more frequently than older patients [50].

### 3.5. Special Situations/Considerations

#### 3.5.1. Coronavirus Disease of 2019 (COVID-19) Related PE

From the beginning of COVID-19 pandemic there was a high suspicion that SARS-CoV2 has a direct connection with venous thrombosis [51]. Studies have provided evidence of a close causal association of COVID19 and PE [51]. The incidence of COVID-19-related PE depends on the clinical setting, the use of thromboprophylaxis and previous history and may be as high as 39.7% [52]. The elevation of D-dimer levels in the COVID-19 population may stem from prothrombotic coagulopathy or pulmonary microvascular thrombosis [52]. 

In patients with COVID-19, the thrombotic lesions were often distributed in the peripheral arteries of the lung, and the total clot burden was lower [53]. VTE events (PE in 1.2% and DVT in 2.3%) during in-hospital stay are increased in the acute phase of COVID19 infection, compared with after discharge hospital incidence of the same events [2]. Further, these VTE events observed in the early phase after disease recovery [2]. VTE is closely link to psychological distress that may be present for months following their hospitalization [54]. Whether patients with COVID-19-related PE present more impaired HRQoL or psychological distress, has not been previously studied in the literature and remains unknown to the date the present review was written.

#### 3.5.2. PE in Patients with Known Mental Illness

There is a direct connection between mental illnesses and PE, proved by several studies. Except of the other comorbidities, mentally ill patients may have additional thromboembolism risk factors that cause PE and may lead to sudden death [55]. Compared with healthy individuals, mental health patients are more vulnerable to PE appearance. Psychotic and bipolar disorders are associated with high PE risk, while Schizophrenia is responsible for most PE cases among those with mental illness [56]. The main characteristics of catatonia is negativism and decreased movement. This, therefore, disrupts venous flow and affects hemostasis [56]. Catatonic and physical restrained patients, stay in bed, even for days, decrease their oral intake (leading to dehydration) and this event may contribute to increase the VTE risk [57]. Anxiety and emotional stress heighten the levels of cortisol and catecholamines, which enforce endothelial friability and damage [57].

Furthermore, obesity, which is common in these patients, may be considered a predisposing risk factor. Various medical treatments for psychotic disorders contribute to the risk of PE. The lower platelet serotonin uptake and the greater serotonin receptor expression stimulates greater platelet activation and, thus, may increase procoagulant properties [57]. Serotonin reuptake inhibitors, lithium, benzodiazepines, especially clozapine and their adverse effects, may contribute to VTE [58]. The majority of studies conclude that the identification and management of predisposing risk factors, along with prophylactic measures, such as those used by medical and surgical patients, are the most important steps to prevent PE in mental illness [57,58,59].

## 4. Future Directions and Conclusions

The current literature supports an impaired HRQoL following PE that improves over time. However, whether this change is clinically meaningful remains to be elucidated. Several instruments have been used to assess HRQoL following the acute event; however, none of them fulfill the current guideline recommendations. Although PEmbQol has been studied previously with remarkable results, the development of new instruments is warranted [35]. PE patients frequently develop symptoms, such as distress, fear of recurrence, anxiety, and they exhibit higher symptom awareness [46]. 

PE sufferers exhibit high levels of anxiety and trauma symptoms [44] with younger patients being at higher risk [40]. However, elderly, previous cardiopulmonary disease, cancer, obesity and persistent symptoms are adversely associated with impaired HRQoL. One may question whether these factors, rather than PE itself, may be associated with worse QoL. One has to take into account that common PE risk factors, such as obesity and cancer, may adversely affect HRQoL and may be associated with mental disorders [60,61]. Studies have identified that comorbidities, such as obesity, active malignancy and cardiopulmonary disease, present independent predictors of HRQoL in the PE population [35]. 

Therefore, one may argue that the impact of PE in QoL and mental may be biased by the potential impact of PE-related risk factors. However, the fact that QoL improves over time takes this concept into question. Additionally, the increased anxiety levels in younger patients, irrespective of comorbidities further challenge this concept. The feeling of upcoming death may result in high levels of post-traumatic stress [40,41]. Depressive symptoms are a common finding that may be present up to 2 years after the event [32]. 

Despite the power of the aforementioned findings, the are some uncertainties that need to be addressed. A major drawback is the lack of prospective assessment of PE-related impairments in mental health and quality of life. Few studies have longitudinally assessed psychological distress and HRQoL in the PE population [46].Whether COVID-19-related PE patients present impaired HRQoL or psychological distress remains unknown and deserves further investigation.

Chronic thromboembolic pulmonary hypertension is a rare complication of PE, which is associated with worse HRQoL [62]. However, published studies that examine HRQoL in long-term (>3 months after diagnosis) do not include CTEPH in the exclusion criteria, although their cohort may report consistent symptoms, such as dyspnea [24]. Therefore, a potential bias cannot be excluded. The design of future studies should take this into account.

Additionally, the effects of different management strategies (i.e., outpatient vs. inpatient therapy, systemic thrombolysis and mechanical measures for high-risk PE) on patients’ mental health have not been examined thoroughly in the literature, and further research is needed. Studies in CTEPH subjects provide evidence for impaired mental health outcomes in subjects not receiving invasive therapy, which, however, were associated with residual pulmonary hypertension [63]. Whether similar findings would be observed in PE is yet to be determined. In the same context, studies have not adequately addressed whether emotional distress is limited or enhanced to patients’ suffering from chronic complications other than CTEPH (i.e., chronic thromboembolic disease/post-PE syndrome), although this seems probable.

Despite the advances in PE management in terms of physical wellbeing, scarce (if any) data exist on interventions aimed at mental distress due to the disease. Current PE guidelines need to include mental health assessment in the follow-up of PE and provide a guide for patients structured follow-up (i.e., at 3, 6, 12 and 24 months after diagnosis), that aims at their psychological status. The identification of groups at risk for mental distress, (e.g., younger patients, patients with cardiopulmonary comorbidities and obese subjects) that could benefit from a targeted approach to reduce the psychological burden, could enable early intervention and, likely, improved outcomes. Fueled by this prospect, well-designed studies are warranted in order to define the optimal follow-up approach (e.g., psychoeducation, empathy and counselling). 

Nonetheless, the increasing body of evidence suggest that health care professionals should not only focus on the physical outcomes of PE but also include their patients’ mental health assessment, since the association of physical and mental health is bidirectional. Health-care professionals may use structured instruments (i.e., PEmbQol) during follow-up and, whenever appropriate, provide timely interventions.

In conclusion, patients with PE are at risk of impaired HRQoL and mental health distress, including anxiety, depression and PTSD, which may be present for years after the event. Most of this knowledge comes from observational studies, and further research is warranted to longitudinally assess the course of mental health impairment as well as to identify groups at risk. The implementation of a structured follow-up aiming to assess the psychological burden following PE seems imperative, and well-designed studies are needed in order to elucidate the optimal follow-up approach.

## Figures and Tables

**Table 1:** The main findings of quality of life in Pulmonary embolism patients. The selection of studies reported in the table is based on the use of adequate QoL measures and the report of clear results of PE in QoL.

Authors	Type	PE Patients	Measurement Tool	Results for QoL	Impacting QoL Predictors
Kahn et al. [29]	Prospective multicenter cohort study	100	SF-36, PEmb-QoL	QoL, dyspnea, walking distance improved at 3 months	Female sex, high BMI, VO2 peak <80% on 1-month cardiopulmonary exercise
Barco et al. [31]	Multinational single-arm trial	576	PEmb-QoL, EQ-5D-5L, Anti-Clot treatment scale	QoL improved at 3 weeks and 3 months	Female sex, high BMI, cardiopulmonary disease
Chuang et al. [34]	Prospective, observational, multicenter study	1399	EQ-5D-5L	QoL improved over time	Bleeding
Valerio et al. [36]	Prospective, observational, multicenter study	620	PEmb-QoL EQ-5D-5L	QoL improved in all dimensions between 3 and 12 months	Female sex, high BMI, cardiopulmonary disease
Keller et al. [24]	Prospective cohort study	101	PEmb-QoL	QoL affected by dyspnea and pulmonary disease	Post-PE impairement
Tavoly et al. [25]	Cross-sectional study	213	EQ-5D-3L	Impraired long term HRQoL (compared with norms)	Poorer performance on 6 min walking test, persistent dyspnea and unemployment.
Klok et al. [35]	Follow-up study, subanalysis	392	SF-36	Impaired QoL (compared with norms)	VTE event, age, obesity and comorbidities
Hernandez-Nino et al. [37]	Mixed-method study	21	Interviews and PEmb-QoL	Impaired QoL	Fear of recurrence, restriction of social life and hobbies

Abbreviations: BMI: body mass index; VO2: oxygen volume; SF-36: short form-36 questionnaire; PEmb-QoL: pulmonary embolism quality of life questionnaire; EQ-5D-5L: EuroQol Group 5 Dimensions 5 Levels; EQ-5D-3L: EuroQol Group 5 Dimensions 3 Levels; HRQoL: health-related quality of life; VTE: venous thromboembolism.

## Data Availability

Not applicable.
